# Identification of a differential metabolite-based signature in patients with late-stage knee osteoarthritis

**DOI:** 10.1016/j.ocarto.2022.100258

**Published:** 2022-04-02

**Authors:** Jason S. Rockel, Mehdi Layeghifard, Y. Raja Rampersaud, Anthony V. Perruccio, Nizar N. Mahomed, J. Roderick Davey, Khalid Syed, Rajiv Gandhi, Mohit Kapoor

**Affiliations:** a Schroeder Arthritis Institute, University Health Network, Toronto, ON, Canada; b Krembil Research Institute, University Health Network, Toronto, ON, Canada; c Program of Genetics & Genome Biology, The Hospital for Sick Children, Toronto, ON, Canada; d Department of Surgery, Faculty of Medicine, University of Toronto, ON, Canada; e Institute of Health Policy, Management & Evaluation, Dalla Lana School of Public Health, University of Toronto, Canada; f Department of Laboratory Medicine and Pathobiology, University of Toronto, Toronto, ON, Canada

**Keywords:** Knee osteoarthritis, Cross-sectional, Metabolomics, Signature, Pathway enrichment

## Abstract

**Objective:**

Multiple disease phenotypes have been identified in knee osteoarthritis (OA) patients based on anthropometric, sociodemographic and clinical factors; however, differential systemic metabolite-based signatures in OA patients are not well understood. We sought to identify differential plasma metabolome signatures in a cross-sectional sample of late-stage knee OA patients.

**Methods:**

Plasma from 214 (56.5% female; mean age ​= ​67.58 years) non-diabetic, non-obese (BMI <30 ​kg/m^2^, mean ​= ​26.25 ​kg/m^2^), radiographic KL 3/4 primary knee OA patients was analyzed by metabolomics. Patients with post-traumatic OA and rheumatoid arthritis were excluded. Hierarchical clustering was used to identify patient clusters based on metabolite levels. A refined metabolite signature differentiating patient clusters was determined based on ≥ 10% difference, significance by FDR-adjusted *t*-test (q-value < 0.05), and random forests importance score ≥1, and analyzed by AUROC. Bioinformatics analysis was used to identify genes linked to ≥2 annotated metabolites. Associated enriched pathways (q ​< ​0.05) were determined.

**Results:**

Two patient clusters were determined based on the levels of 151 metabolites identified. Metabolite signature refinement found 24 metabolites could accurately predict cluster classification within the sample (AUC ​= ​0.921). Fifty-six genes were linked to at least 2 ​KEGG annotated metabolites. Pathway analysis found 26/56 genes were linked to enriched pathways including tRNA acylation and B-vitamin metabolism.

**Conclusion:**

This study demonstrates systemic metabolites can classify a cross-sectional cohort of OA patients into distinct clusters. Links between metabolites, genes and pathways can help determine biological differences between OA patients, potentially improving precision medicine and decision-making.

## 1 Introduction

Osteoarthritis (OA) is a degenerative joint disorder with local joint involvement including joint degeneration, synovitis and bone formation [1], in addition to changes in systemic factors including cytokines, microRNAs and metabolites [[2], [3], [4], [5]]. In addition to genetics [6], sociodemographic and anthropometric variables such as age, sex and body mass index (BMI) have been identified as major contributors to the onset and progression of OA [7,8]. Furthermore, multiple phenotypes of OA have been described that include major clinical features such as inflammation, low muscle mass, pain and obesity [9,10].

Circulating factors have potential biomarker and prognostic capabilities for OA. For instance, metabolites have emerged as being capable of identifying patients with OA from healthy individuals [11], classifying different metabolite phenotypes in OA patients [12], and differentiating individuals more likely to respond to surgical intervention from non-responders [13], important for clinical decision making. However, some patient factors, including diabetes and obesity, also impact circulating metabolite levels [14], complicating the discovery of metabolite signatures given that these two conditions are not uncommon in patients with OA. Furthermore, other patient variables, such as age and sex, also independently contribute to differences in the circulating metabolome [15,16], potentially complicating metabolite signature discovery.

As a variety of anthropometric, demographic and clinical factors can modify circulating metabolites, in this initial cross-sectional study, we sought to interrogate, in an unbiased fashion, whether there were unique signatures of plasma metabolites, identified by metabolomics, that could differentially classify participants from a sample of 214 non diabetic, non-obese, late-stage knee OA patients.

## 2 Methods

### 2.1 Patient sample

Patients were selected, under informed consent, from the Longitudinal Evaluation in the Osteoarthritis Program-OA (LEAP-OA) cohort (REB# 14–7592, 16-5759be). Patients had radiographic Kellgren-Lawrence grade (KL)3/4 primary knee OA (as diagnosed by an orthopaedic surgeon) and were scheduled to undergo total knee arthroplasty (TKA) surgery (late-stage knee OA). Patients were non-diabetic and non-obese (BMI <30) based on self-reported questionnaire and clinical assessment. Patients with post-traumatic OA and rheumatoid arthritis were excluded from the study. Patients had a minimum of 8 ​h solid food fasting and 6 ​h fluid fasting. The self-reported questionnaire, completed within the 3 months prior to scheduled surgery, was used to acquire data on sociodemographic (ethnicity), anthropometric (age, sex), and health-related data, including 23 comorbidities and medication use (over the counter, non-steroidal anti-inflammatories, narcotics, muscle relaxants, anti-depressants, or neuroleptics). BMI of each patient was calculated using measured height and weight. The distribution of sociodemographic and anthropometric data for the sample are shown in Table 1 and Supplementary Table 1.

**Table 1:** Demographics of late-stage knee OA patients within the LEAP-OA cohort with BMI <30 and without patient-reported diabetes.

Sex	n	Mean Age (years, 95% CI)	Mean BMI (kg/m2, 95% CI)	Females (n, %)
All	214	67.58 (66.45, 68.71)	26.25 (25.93, 26.58)	121 (57)
Males	93	67.72 (65.95, 69.49)	26.66 (26.23, 27.08)	0 (0)
Females	121	67.47 (65.99, 68.95)	25.94 (25.47, 26.41)	121 (100)

Proportion of females in the cohort (P = 0.0647), and comparisons of age (P = 0.8331) and BMI (P = 0.0697) between males and females, were determined by two-tailed binomial and Mann-Whitney tests, respectively. BMI, body mass index; CI, confidence intervals.

### 2.2 Metabolomics

Blood was collected at time of surgery in K_2_EDTA-plasma tubes. Plasma was separated by centrifugation of whole blood at 4000 ​rpm for 10 ​min at 4 ​°C (Eppendorf 5810 ​R Centrifuge). Plasma was aliquoted into cryovials and protease inhibitors were added. Plasma was subsequently flash frozen and stored in liquid nitrogen. Metabolomics profiling of 188 metabolites in the 214 human plasma samples was performed at The Metabolomics Innovation Centre (Calgary, Alberta) by Liquid Chromatography (LC)/Mass Spectrometry (MS)/MS using Biocrates AbsoluteIDQ p180 kit following manufacturer's directions. Absolute IDQ-coupled MetIDQ software (Biocrates Life Sciences AG, Austria) was used for metabolite quantification and batch correction.

### 2.3 Hierarchical clustering

Unsupervised agglomerative hierarchical clustering was performed using raw metabolite data in SciPy package v1.5.2 in Python. The algorithm starts by finding the metabolite data of patients that are closest to each other using the Ward variance minimization algorithm. The next step was to join the newly-formed two-patient cluster to the next nearest patient, which in turn results in a new cluster. This approach continued in a bottom-up manner until all the patients were clustered. The resulting clusters are shown as a dendrogram where the vertical height shows the Ward distances between patients.

To further confirm the accuracy of the clustering, we used betadisper test [multivariate homogeneity of groups dispersions (variances)], which tests whether two or more groups are homogeneously dispersed in relation to their group centroid. This test can be done to see if one group has more compositional variance than another. Betadisper (vegan v2.4–2 in R) first calculated the average distance of group members to the group centroid in multivariate space (generated by a distance matrix). An ANOVA was then done to test if the dispersions (variances) of groups were different.

### 2.4 Statistical analyses of the cross-sectional sample

Statistical analyses of differences in the sample were performed using Graphpad v8.4 (Prism Software LLC). The deviation of the proportion of females and males in the cohort from 50% was evaluated by a two-tailed binomial test. Differences in age and BMI between sexes were determined by two-tailed Mann-Whitney U-tests. P ​< ​0.05 was considered a statistically significant difference/deviation. After identification of the two clusters, Fisher's exact test was used to determine whether there were any significant differences in the distribution of age, sex, BMI, comorbidities and medication use between clusters, with p ​< ​0.05 considered significant.

### 2.5 Metabolite signature identification

After hierarchical clustering, metabolite concentrations were normalized by sum, log-transformed, and scaled using the Pareto approach (mean-centered and divided by the square root of the standard deviation of each variable; MetaboAnalyst's Biomarker Analysis module). Significant differences in metabolite levels between clusters or patients with anthropometric, sociodemographic, comorbidities or drug use measures identified to be significant by Fisher's exact test were determined by *t*-test (FDR-adjusted p (q) ​< ​0.05 was considered significant). Metabolites with q ​< ​0.05 were selected and those that had a difference ≥10% were retained. Overlap of metabolites between significant metabolites determined in clusters and covariates with significantly different distribution between clusters was also determined. The most important metabolites capable of distinguishing individual clusters were identified using Random Forest with cross validation implemented in MetaboAnalyst v4.0 (Multivariate Exploratory ROC Analysis section of Biomarker Analysis module [17]). Metabolites with q ​< ​0.05, difference ≥10%, and feature importance ≥1 were selected for further analysis. The metabolite signature was tested in the biomarker analysis module in Metaboanalyst to determine AUC and predictive accuracy across 100 cross-validations. The significance of the AUC curve and prediction accuracy was determined across 1000 permutations. P ​< ​0.05 was considered significant.

### 2.6 Gene-metabolite network modeling and pathway analysis

Gene-metabolite interaction networks were inferred for the metabolite signature identified using MetaboAnalyst's Network Explorer module. The networks help identify the respective gene targets of each metabolite. This tool only uses highly confident gene-metabolite associations from STITCH [18]. From this network, the genes (i.e. metabolite-linked targets) with degree ≥2 were extracted for the subsequent pathway analysis. Pathway analysis and enrichment was performed using pathDIP [19], which integrates core pathways from 20 source databases (pathDIP version 4.0.21.2; Database version 4.0.7.0). Significantly enriched pathways were identified with pathDIP parameters set as extended pathway associations to integrate core pathways with a protein-protein interaction (PPI) set containing experimentally detected PPIs. The minimum confidence level for predicted associations was set at 0.99. Significantly enriched pathways were considered as those with Benjamini-Hochberg q ​< ​0.05.

## 3 Results

### 3.1 Metabolites alone identify two main clusters of late-stage knee OA patients

**Fig. 1:**
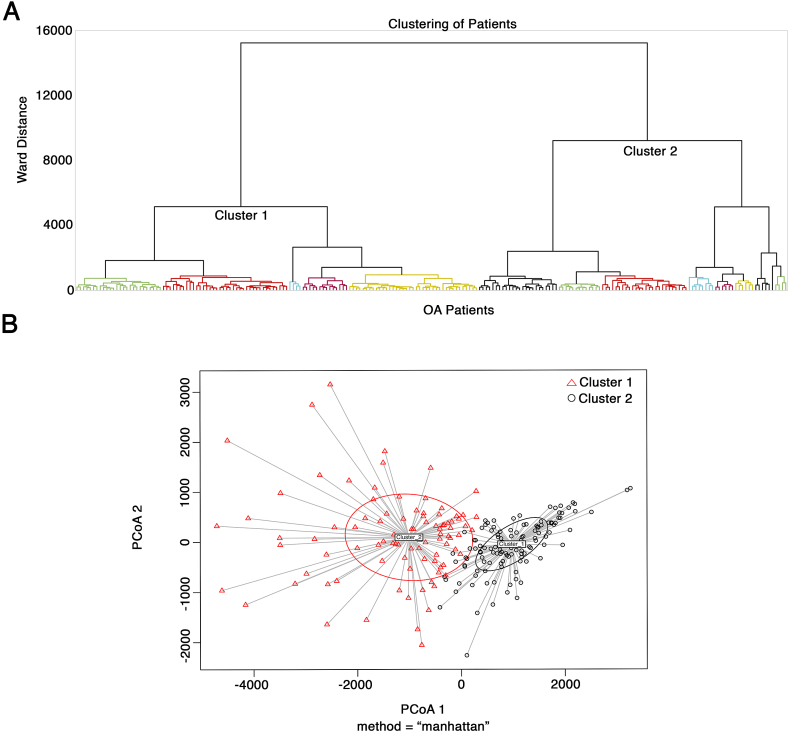
The cross-sectional OA patient population can be divided into two clusters based on metabolites. (**A**) Hierarchical clustering of OA patients based on the detected levels of metabolites. Individual patients are separated into two distinct overarching clusters, based on Ward distance. (**B**) The distance of individuals in each cluster to the cluster centroid, as calculated using the betadisper method, shows the distinction between the two clusters obtained from hierarchical clustering. Individual patients are represented by black circles (cluster 1) or red triangles (cluster 2).

Using an unsupervised approach, we sought to determine if we could identify clusters of late-stage knee OA patients using only metabolite levels. We investigated a cross-sectional sample of non-obese, non-diabetic OA patients (Table 1; Supplemental Table 1) from the LEAP-OA cohort to isolate metabolites linked to clusters of OA patients from the sample and not metabolically-relevant covariates. Representation of males (N ​= ​93) and females (N ​= ​121) was not significantly different (p ​> ​0.05 by binomial test). Age and BMI of the sexes was also not significantly different (p ​> ​0.05 by Mann Whitney U tests). Using metabolomics screening of patient plasma, we identified 151 of a possible 188 individual metabolites (Supplemental Table 2). Using only metabolite levels, unsupervised hierarchical clustering of the OA patients identified two major clusters, namely cluster 1 and cluster 2 (Fig. 1A; Table 2; Supplemental Table 3). Cluster 1 contained 121 patients with cluster 2 containing 93 total patients (Table 2; Supplemental Table 3). Clustering was further confirmed using betadisper test, which distinguishes the two clusters after plotting the first two principal components (Fig. 1B).

**Table 2:** Summary of the distribution of patient characteristics based on cluster classification.

Cluster	n	Females (n)	Males (n)	Mean Age (years, 95% CI)	Mean BMI (kg/m2, 95% CI)	Comorbidities (n, ≤ 5 out of 23)	Daily Medication Use (n, ≥ 1)
1	121	74	47	66.74 (65.36, 68.13)	26.14 (25.7, 26.58)	111	64
2	93	47	46	68.67 (66.79, 70.54)	26.39 (25.91, 26.88)	81	53

Distribution of sexes, ages (continuous or 2 quantiles based on median), BMI (continuous or 2 quantiles based on median), comorbidities, or at least 1 daily medication use (median ​= ​0) was not significantly different between clusters based on two-sided Fisher's exact tests or two-tailed Mann-Whitney U-tests (P ​> ​0.05). See Supplemental Table 1. BMI, body mass index; CI, confidence interval.

### 3.2 Identified clusters are largely independent of demographic, anthropometric or clinical variables

We next tested if there was a deviation in the proportion of patients divided into the two clusters by demographic, anthropometric, or clinical variables (Table 2; Supplemental Table 1; Supplemental Table 3). Variables tested included age (continuous or split into 2 quantiles based on median of 67 years), sex, BMI (continuous or split into 2 quantiles based on median of 26.51 ​kg/m^2^), ethnicity (split into 2 quantiles based on patient-reported as Caucasian vs. non-Caucasian), 23 individual patient-reported comorbidities, patients with >5 reported comorbidities (median of the population), and 7 categories of patient-reported medication use [each individual medication use (6 total) split into 2 quantiles based on never/sometimes use vs. any daily medication use]. Overall, 36 different comparisons were conducted. The only significant difference in the proportions of patients divided between the two identified clusters was by daily narcotic use, which was more prevalent in cluster 2 compared to cluster 1 (p ​= ​0.0041 by Fisher's exact test).

### 3.3 Identification of a differential metabolite network linked to clusters

**Fig. 2:**
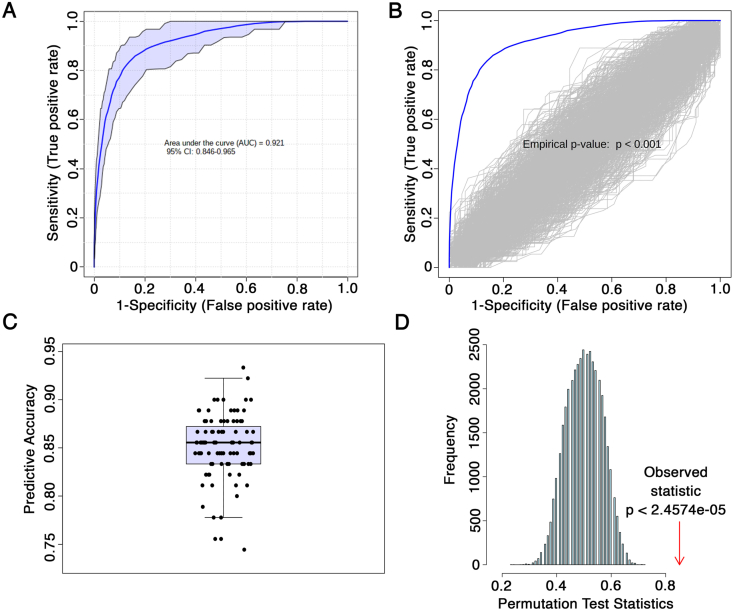
Differential cluster-based metabolites can form a refined signature of 24 metabolites with excellent cluster classification ability. (**A**) Mean AUROC curve from 100 cross-validations of the 24 metabolite signature differentiating cluster 1 from cluster 2 in the cross-sectional OA population. (**B**) Empirical AUROC curve analysis based on 1000 permutations. (**C**) Predictive accuracy across 100 cross-validation of the 24 metabolite signature differentiating cluster 1 from cluster 2 in the cross-sectional OA population. (**D**) Significance of predictive accuracy based on 1000 permutations.

We first identified metabolites whose levels were significantly different between the two clusters. We determined that 46 metabolites were significantly different between clusters (q ​< ​0.05) by at least 10% based on their levels. We next determined which metabolites were most important at differentiating the two clusters. Using random forest modeling with cross-validation, important feature selection indicated 32 metabolites had a score ≥1. Overall, 24 metabolites were common between the two lists, which were used as our metabolite signature moving forward (Supplemental Table 4). The metabolite signature identified consisted of 7 ​amino acids, 2 biogenic amines, 12 phosphatidylcholines, 1 lysophosphatidylcholine, 1 carnitine and 1 sphingomyelin (Table 3). No metabolites were significantly modified in daily narcotic users compared to other individuals (q ​> ​0.05), suggesting no significant influence of this group of patients on overall metabolite levels between clusters. The signature of 24 metabolites was able to separate the sample into the two clusters with excellent classification accuracy (Mean AUC ​= ​0.921; mean predicted accuracy of 85.1% across 100 cross-validations with Random forests modeling; AUC curve empirical p ​< ​0.001; predictive accuracy p ​< ​0.0001 with 1000 permutations; Fig. 2).

**Table 3:**
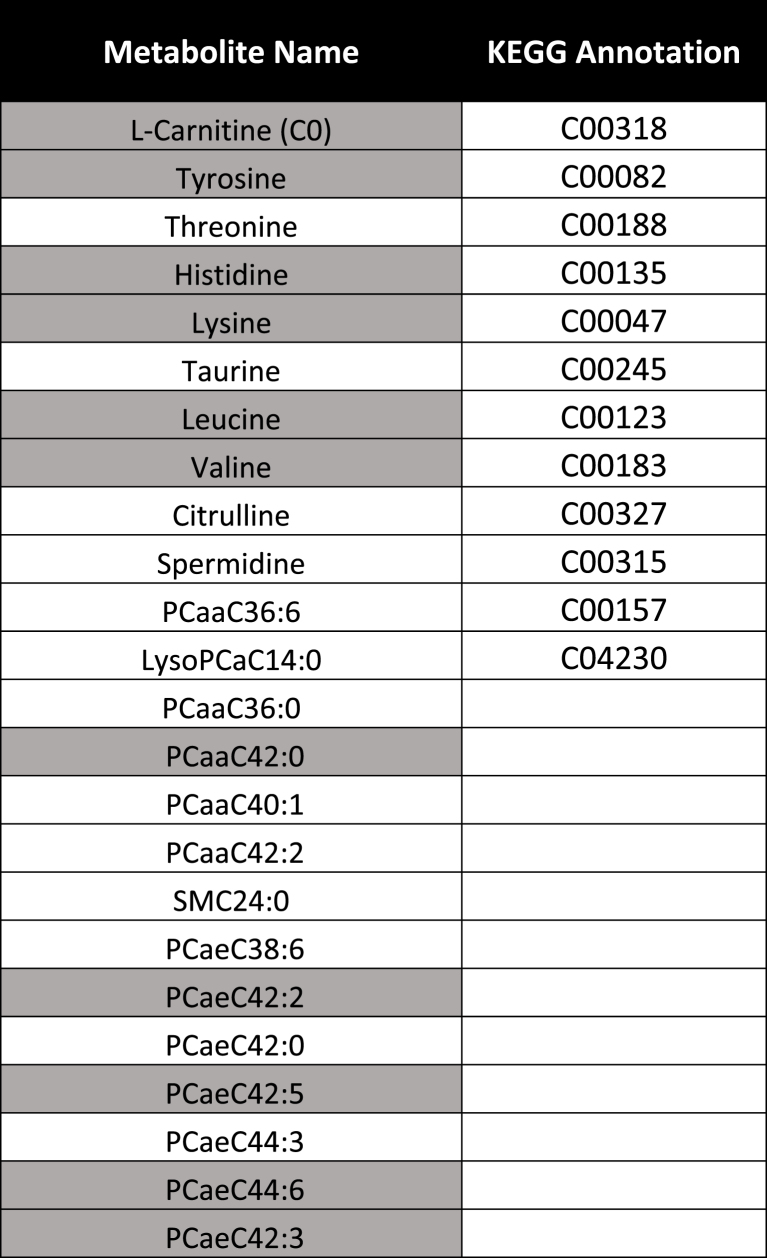
Refined metabolite signature and annotated KEGG compound identities. Grey cells reflect metabolites also identified in metabolite signatures from Zhang et al. [12].

**Fig. 3:**
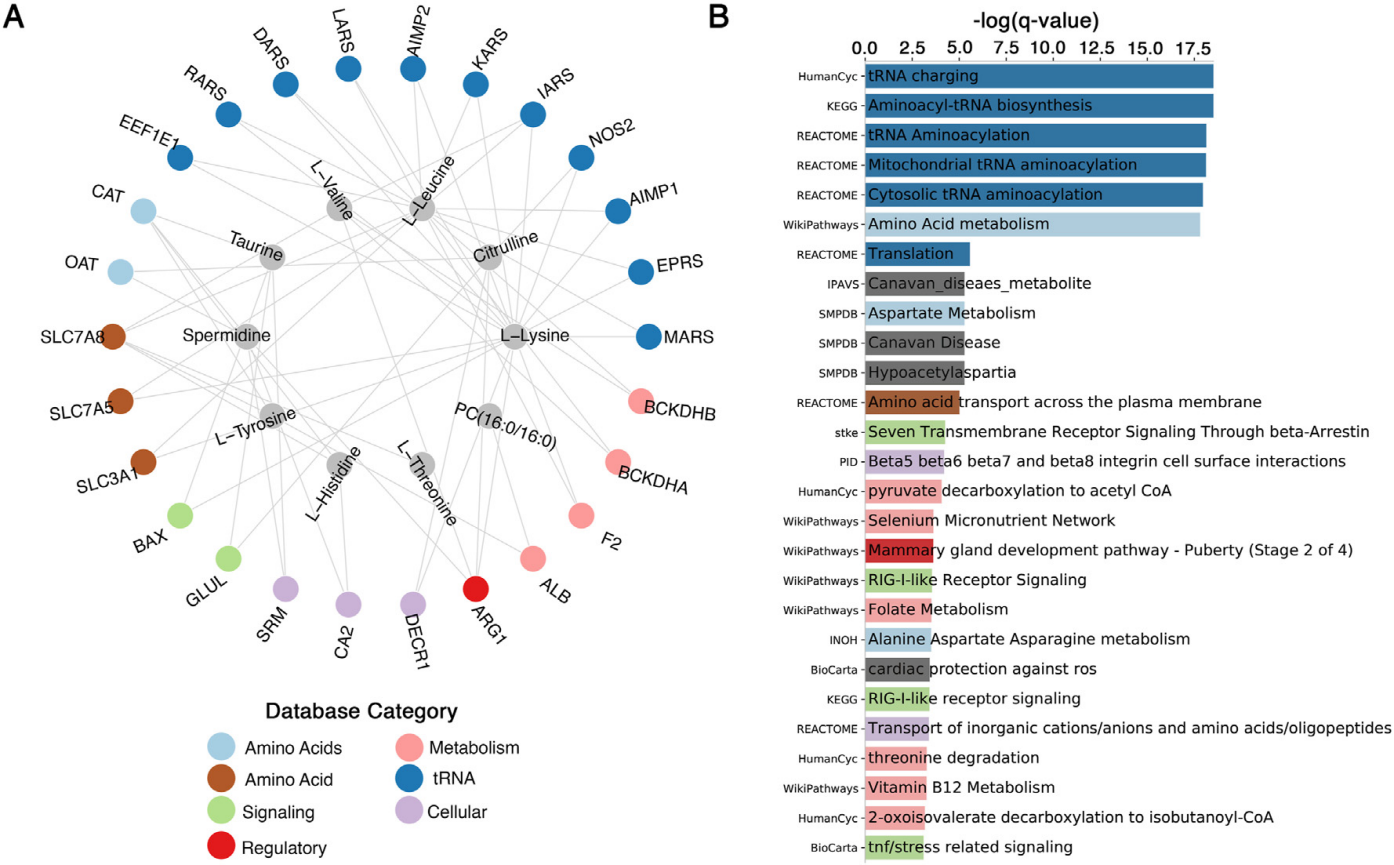
The refined metabolite signature is linked to a gene network with putative enriched pathways. (**A**) Gene-metabolite network of 26 genes linked to enriched pathways and annotated metabolites as part of the refined 24 metabolite signature. (**B**) The list of enriched pathways organized by FDR-corrected (Benjamini-Hochberg) *P*-value (q). (**A&B**) Genes in the network and enriched pathways are color coded based on the broad functional descriptions of the pathways. In cases where a gene was enriched in more than one pathway, the color for the pathway with the lowest q-value was chosen. Enriched pathways in grey are associated with metabolite-linked genes but are not top enriched pathways for any individual gene, and thus are not distinguished by a broader database category.

We next generated a gene-metabolite network using our identified metabolite signature. Of the 24 individual metabolites, only 12 were annotated in KEGG, with the single sphingomyelin (SM 24:0) and 11/12 phosphatidylcholines (PCs) not annotated (PCaa36:6 was annotated; however, it was annotated as the common phosphatidylcholine PCaa36:0). Of the 12 annotated metabolites, only LysoPC14:0 was not linked to any genes. The remaining metabolites were linked to 429 individual genes by at least one degree (Supplemental Table 5). To identify key genes most linked to the metabolite signature, we selected genes that were linked to a minimum of 2 metabolites for pathway analysis. A total of 56 genes were linked to at least 2 of the 11 annotated metabolites as part of our identified signature. Of the 56 genes, 26 were annotated to significantly enriched pathways (Benjamini-Hochberg q ​< ​0.05), as determined by pathway analysis in Pathdip (Fig. 3; Supplemental Table 6). Top enriched pathways were related to tRNA acylation. Interestingly, B-vitamin metabolism [B12 and folate (B9)] was also enriched. Overall, this novel metabolite signature suggests underlying metabolic phenotypes in OA patients independent of multiple covariates.

## 4 Discussion

Through statistical modeling of cross-sectional data from a sample of late-stage knee OA patients, we found two patient clusters that could be classified by plasma levels of 24 metabolites, independent of demographic and anthropometric covariates. In addition, we determined a metabolite-gene network and enriched pathways which could be modified between the two clusters based on the differential metabolite signature.

Multiple studies have identified connections between metabolite levels and patient variables including age, sex, comorbidities (diabetes), and BMI [2,[14], [15], [16]]. We limited the potential impact of diabetes and obesity by focusing exclusively on non-diabetic and non-obese patients. Furthermore, we found no significant impact of any clinical, sociodemographic or anthropometric variables on the differential metabolite signature identified, including age, sex, BMI, comorbidities, and drug use. This suggests that the classification of individuals into the two identified clusters may be due to distinguishing biological features unique to subgroups of late-stage knee OA patients. However, given the marginally non-significant difference in age between the two identified clusters (dichotomous p ​= ​0.07; continuous p ​= ​0.10), we cannot exclude that part of the clustering and associated metabolite levels may be impacted by age.

A previous study of 80 late-stage knee OA patients found two major clusters defined by metabolites alone [12]. One of the major clusters contained 69/81 participants and could further be split into two secondary clusters. Interestingly, the authors reported carnitine C0 as a major contributor distinguishing the two major clusters, consistent with the metabolite identified as part of the differential metabolite signature in our study. Furthermore, 5/7 ​amino acids and 5/12 phosphatidylcholines were also common to the signature identified in the current study (Table 3 metabolites highlighted in grey) compared to that from significant metabolites and VIP scores separating subclusters of the secondary groups from the aforementioned study. These 11 metabolites provided good classification in our sample (AUC ​= ​0.739 by random forests modeling), suggesting that they may be important for distinguishing underlying metabolic differences in patients with late-stage knee OA. Differences in amino acids, biogenic amines and other PC/lysoPC analogues not common amongst the metabolites signatures from these studies could simply be due to differences between the samples examined, as the current study excluded obese and diabetic individuals, whereas the aforementioned study did not.

Of the pathways identified as being enriched by metabolite-gene linkages based on the identified metabolite signature, tRNA charging/aminoacylation was the most significantly enriched process, which included aminoacyl tRNA synthetases (ARS) iRARS, DARS, LARS, KARS, IARS, EPRS, and MARS, genes linked to amino acids identified in our identified metabolic signature. Although this enriched pathway may simply be related to the large number of amino acids in the metabolite signature identified, tRNA aminoacyl synthetases have various extracellular and intracellular roles. KARS can be secreted from cells and act as a cytokine to increase cytokine release from macrophages, including TNFα [20]. Other tRNA aminoacyl synthetases and associated complex proteins, such as AIMP1 and AIMP2, can modulate inflammation, apoptosis and gene expression (as previously reviewed [21]), while downregulation of AIMP1 in chondrocytes promotes anabolic cell activity by modulating TGFβ signaling [22]. Interestingly, vitamin B9 and B12 metabolic pathways were also found to be enriched by analysis of the metabolite-gene linkages. A clinical trial in hand OA found that supplementation with these two vitamins improves hand function [23], while a B-vitamin complex that includes B12 improves responses to NSAID treatment in late-stage knee OA patients [24]. Thus the metabolite-gene network identified has links to possible differentially regulated mechanisms in patient clusters; however, modification of enriched pathways in patient clusters requires thorough investigation.

We studied systemic metabolite levels in this cross-sectional sample of patients with OA. Correlations between local (knee synovial fluid) and systemic (serum) metabolite levels are modest [25]. However, changes in both synovial fluid and plasma metabolomes have been linked to changes in radiographic grading of OA [26,27], suggesting that OA progression is linked to metabolite changes at the local and systemic levels. To our knowledge, no published studies have linked synovial fluid metabolites to pain among patients with OA; thus further analyses of the link between the local metabolome and OA symptoms are needed. In contrast, a plasma metabolite signature has been linked to pain response in patients who received TJA after one year [13]. Together, these suggest that the systemic metabolome may, in time, show utility in diagnostic or predictive models for OA disease and symptoms in patients with OA; however, a direct comparison of metabolites in synovial fluid and the circulation is needed to determine which fluid metabolome best correlates/associates with disease and symptom changes or outcomes.

In the current study, the sample of patients studied was recruited only after diagnosis of late-stage knee OA and not longitudinally tracked from early stages. Thus, we were unable to retrospectively evaluate whether the two identified clusters had different trajectories of pain or function leading up to TKA, or time to TKA. However, as up to 34% of patients undergoing TKA for OA do not generally achieve a clinically-relevant reduction in pain after surgery [28], the identified metabolite signature could be evaluated as a predictive factor for TKA outcome, the subject of a future study.

This study is not without limitations. Comorbidities, including diabetes, were evaluated solely based on patient reported questionnaires; thus blood sugar levels and other biomarkers of reported diseases are likely to be more valuable in future studies to confirm diagnosis for study exclusion. A more sedentary lifestyle can also impact the circulating metabolism [29], a potential confounding factor for which data were not available for in this cross-sectional sample. Additionally, available annotations of metabolites for gene linkages are not very diverse. Metabolite databases have incomplete annotations, with some completely missing (SM24:0), and others grouped based on classification (such as phosphatidylcholines). This reduces the amount of information that can be gathered from individual metabolites. Evidence suggests that not all metabolite analogues are equally metabolized by target enzymes. For instance, the activity of autotaxin, the enzyme that catalyzes the production of lysophosphatidic acid from LysoPCs, depends on structure and concentration of its substrates [30,31]. Thus, annotating metabolites based simply on classification may not be ideal. Further metabolite annotations and gene linkages based on concentration are needed to fully appreciate pathways linked to metabolite signatures. Additional cohorts of individuals with late-stage radiographic KL3/4 primary OA, who are also non-diabetic and non-obese, are also needed to validate if the 24 metabolite signature can classify individuals into metabolite-based clusters similar to that observed in this study. Finally, given all patients were late-stage knee OA, the clinical implications of our findings may be limited to correlation with clinical symptom profiles (e.g. pain severity) or surgical response. For example, despite the general effectiveness of TKA for knee OA, not all patients respond to surgery [28]. Several sociodemographic and anthropometric factors have only been able to explain approximately 30% of the variance of the response to surgery [32,33], suggesting a contribution of the underlying biological differences in patients with OA. This degree of variation in outcome warrants further study to determine whether the identified metabolite signature from this unique subset of OA patients can predict outcomes of surgery.

Overall, we determined two metabolite-based OA phenotypes from a cross-sectional sample of late-stage knee OA patients largely defined by a differential signature of 24 metabolites. The metabolite-gene network and enriched pathways identified from this signature may help elucidate and explain differences with respect to disease mechanisms, symptoms and post-surgical responses in late-stage, non-diabetic, non-obese patients with OA, helping to inform future precision medicine approaches.

## Author contributions

**JR**, **ML**, **YRR**, **RG** and **MK** were involved in the conception and design of the study. **JR** and **ML** performed statistical and bioinformatical analyses. **RR, AP**, **NM**, **RD**, **KS**, and **RG** collected patient samples and/or data. **KP** and **AW** were involved in processing, storage and tracking of samples and patient data. All authors were involved in drafting, revising and approving the final version of the manuscript.

## Role of funding sources

This work is supported by the grants to MK by the 10.13039/501100001804Canada Research Chairs Program (950–232,237), Canadian Institute of Health Research Operating Grants (FRN: 377,743), Tony and Shari Fell Platinum Chair in Arthritis Research, and Campaign to Cure Arthritis via the Toronto General and Western Foundation. AVP is supported by an award from the Arthritis Society (STAR-20-0000000012). The funders had no role in study design, data collection and analysis, decision to publish, or preparation of the manuscript.

## Declaration of competing interest

The authors declare no conflicts of interest related to this manuscript.
